# Evaluation of the EtOAc Extract of Lemongrass (*Cymbopogon citratus*) as a Potential Skincare Cosmetic Material for Acne Vulgaris

**DOI:** 10.4014/jmb.2201.01037

**Published:** 2022-04-21

**Authors:** Chowon Kim, Jumin Park, Hyeyoung Lee, Dae-Youn Hwang, So Hae Park, Heeseob Lee

**Affiliations:** 1 Department of Food Science and Nutrition, College of Human Ecology, Pusan National University, Busan 46241, Republic of Korea; 2 Division of Applied Bioengineering, Dong-Eui University, Busan 47340, Republic of Korea; 3 Department of Biomaterials Science, Pusan National University, Miryang 50463, Republic of Korea; 4 Longevity & Wellbeing Research Center, Laboratory Animals Resources Center, College of Natural Resources and Life Science, Pusan National University, Miryang 50463, Republic of Korea

**Keywords:** Lemongrass, EtOAc extract, antioxidant activity, antimicrobial activity, skincare, *Cutibacterium acnes*

## Abstract

This study evaluated the biological properties of lemongrass (*Cymbopogon citratus*) extracts. The EtOAc extract of lemongrass had DPPH, TEAC, and nitric oxide-scavenging activity assay results of 58.06, 44.14, and 41.08% at the concentration of 50, 10, and 50 μg/ml, respectively. The EtOAc extract had higher elastase and collagenase inhibitory activities than the 80% MeOH, n-hexane, BuOH, and water extracts and comparable whitening activity toward monophenolase or diphenolase. Also, the EtOAc fraction had higher lipase inhibitory and antimicrobial activities against *Cutibacterium acnes* among extracts which is known to an important contributor to the progression of inflammatory acne vulgaris, and an opportunistic pathogen present in human skin. Total phenolic and flavonoid concentrations in the EtOAc extract were 132.31 mg CAE/g extract and 104.50 mg NE/g extract, respectively. Biologically active compounds in lemongrass extracts were analyzed by LC-MS. This study confirms that lemongrass extracts have potential use as cosmetic skincare ingredients. Thus, lemongrass can be considered a promising natural source of readily available, low-cost extracts rich in antioxidant, skincare, and antimicrobial compounds that might be suitable for replacing synthetic compounds in the cosmeceutical industry.

## Introduction

Lemongrass (*Cymbopogon citratus*) is a fragrant plant with a lemon scent belonging to the Gramineae family and is cultivated mainly in tropical regions such as India and Malaysia, but recently, due to climate changes and the increased use of herbs, lemongrass has been cultivated in Korea [1-3]. Lemongrass continues to be used as a folk remedy to treat coughs, epithelial diseases, flu, pneumonia, headache, leprosy, malaria, gingivitis, and vascular disorders [4], and in some countries, to treat acne, pimples, and blackheads or even lice and dandruff [5]. *Cymbopogon citratus* has been reported to contain a variety of active compounds, including citral, chlorogenic acid, luteolin, p-coumaric acid, apigenin, and caffeic acid [6, 7].

Previous studies have identified and isolated phenolics from lemongrass that function as antioxidants and have several positive health effects [8, 9]. Moreover, it has been demonstrated that extracts of *C. ciratus* leaves exhibit antimicrobial activity against many kinds of microorganisms [10-12]. Compounds such as phenolics and flavonoids are widely distributed in plant products. These polyphenols comprise a large group of natural compounds with broad biological activities and applicabilities due to their antioxidant, antitumor, antiviral, anti-inflammatory, antibiotic, and allelopathic effects [13].

DPPH radical assays have been widely used to test the free radical scavenging abilities of various natural products, and DPPH is considered a model compound for determining the free radical scavenging abilities of lipids [14]. ABTS and DPPH assays are widely used to assess the antioxidant properties of natural products. Both are spectrophotometric techniques based on the quenching of stably colored radicals and can be used to determine the radical scavenging abilities of antioxidants even in complex biological mixtures such as plant or food extracts [15]. Despite the beneficial effects of NO·, its contribution to oxidative damage is becoming increasingly evident. NO· can react with superoxide to form the peroxynitrite anion, a strong oxidant that can decompose to produce ·OH and NO_2_ [16,17], and the NO· released by sodium nitroprusside has can form NO^+^ and chemically modify many cellular components.

Acne vulgaris is a common chronic inflammatory skin disease, characterized by lesions such as comedones, pustules, cysts, and nodules. The causes of acne vulgaris have not been elucidated, but four major factors have been identified, that is, follicular hyperkeratinization, excess sebum secretion, colonization by *Cutibacterium acnes*, and immunoinflammatory responses [18]. *Cutibacterium acnes* is a gram-positive, lipolytic, anaerobic microorganism, an important contributor to the progression of inflammatory acne vulgaris, and an opportunistic pathogen present in the sebaceous follicles of human skin [19-21].

This study was performed to evaluate the biological properties of lemongrass extracts toward anti-oxidant, anti-anging, anti-whitening, and *C. acnes* inhibitory activities. Also, functional compounds were identified to develop lemongrass as a potential skincare cosmetic material.

## Materials and Methods

### Sample Preparation

The lemongrass was obtained from Namwon-si, Jeollabuk-do, Korea, washed, freeze-dried (Eyela FDU‐2100, Tokyo Rikakikai Co. Ltd., Japan), powdered, and stored -20°C until needed.

### Organic Solvent Fractions

The lemongrass powder (10 g) was extracted overnight with 200 ml of 80% methanol (MeOH) (v/v) using a shaking incubator (VS-8480, Vision Scientific, Korea). After filtration using Whatman No. 2 filter paper (Whatman, UK), the filtrate was evaporated and freeze-dried. The 80% MeOH extracts (10 g) were dissolved in 200 ml of distilled water and fractionated with n-hexane, ethyl acetate (EtOAc), and butanol (BuOH). The lemongrass extracts were stored at -20°C until needed.

### Strains and Culture Condition of Microorganism

*Cutibacterium acnes* (KCTC 3314) was purchased from the Korean Collection for Type Cultures (KCTC), cultivated in brain heart infusion broth (Difco Laboratories, USA) containing agar powder (Junsei Chemical Co., Ltd., Japan), and incubated anaerobically in an anaerobic jar (BD Biosciences, USA) with a CO_2_ gas pak (OxoidTM AnaeroGenTM 2.5L Sachet, Thermo Fisher Scientific Inc., USA) at 37°C for 48 h in an incubator (Jisico Co., Ltd., Korea).

### DPPH Radical Scavenging Activity

DPPH (1,1-diphenyl-2-picrylhydrazyl) radical scavenging assays of lemongrass extracts were performed using a modified Blois method [22]. Briefly, 60 μM of DPPH solution in 95% ethanol was dissolved and filtered, and 100 μl of DPPH solution and 100 μl of diluted sample solution were mixed and incubated at room temperature for 30 min in the dark. Absorbances were measured at 540 nm.

### ABTS Radical Scavenging Activity

Total antioxidant activity was measured using an ABTS (2,2′-azono-bis-3-ethylbenzthiazoline-6-sulphonate) decolorization assay [23]. A solution of 7 mM of ABTS in distilled water and 2.45 mM potassium persulfate were mixed and held at room temperature for 12 h in the dark. The ABTS radical cation solution was then diluted with 5 mM PBS (pH 7.4) and adjusted to an absorbance of 0.70 ± 0.02 at 734 nm. 990 μl of the diluted solution and 10 μl of sample were then mixed and allowed to react for 6 min in the dark. Absorbances were measured using a spectrophotometer at 734 nm. Ascorbic acid was used as the positive control.

### Nitric oxide (NO) Scavenging Activity

The modified Jaiswal method was used to evaluate nitric oxide (NO) scavenging activity [24]. Griess reagent was composed of 1% sulfanilamide (w/v), 5% phosphoric acid (v/v), and 0.1% N-(1-naphthyl) ethylenediamine dihydrochloride (w/v). 100 μl of samples dissolved in distilled water, 1 ml of phosphate buffer (pH 7.4), and 400 μl of 10 mM sodium nitroprusside were mixed, and then incubated at room temperature for 2 h 30 min. Subsequently, 200 μl of these mixtures were added to 200 μl of Griess reagent and reacted for 30 min. Absorbances were measured at 540 nm.

### Elastase Inhibitory Assay

The elastase inhibitory assay was conducted using the modified Cannell method [25]. *N*-succinyl-(L-Ala)_3_-p-nitroanilide (Sigma Chemical Co., USA) was used as a substrate and dissolved in buffer solution to a concentration of 2.9 mM. Briefly, 15 μl of 0.5 U/mL elastase (Sigma Chemical Co.) dissolved in buffer (0.1 M Tris-HCl; pH 8.0), 100 μl of same buffer, 20 μl of substrate, and 15 μl of sample were mixed and incubated at 37°C for 30 min. One hundred μg/ml of ursolic acid was used as the positive control. To evaluate elastase inhibitory activity, absorbance was measured at 420 nm using a micro-plate reader (Tecan Sunrise, Tecan, Switzerland).

### Collagenase Inhibitory Assay

Collagenase inhibitory activity was determined using a slight modification of the method devised by Wünsch and Heindrich [26]. 4-Phenylazobenzyloxycarbonyl-Pro-Leu-Gly-Pro-D-Arg (Sigma Chemical Co.) at 0.3 mg/ml was used as a substrate. Collagenase (Clostridium; Sigma Chemical Co.) was dissolved in buffer solution (100 mM Tris-HCl buffer (pH 7.5) containing 4 mM CaCl_2_) to 0.2 mg/ml. 100 μl of each sample solution, 250 μl of substrate solution, and 150 μl of collagenase were then mixed and reacted at 25°C for 20 min. The reaction was stopped by adding 5 ml of EtOAc and then 500 μl of 6% citric acid (Sigma Chemical Co.) was added to separate reaction mixtures. Epigallocatechin gallate (16.5 μg/ml) was used as a positive control. Supernatant absorbances were measured at 320 nm using a spectrophotometer.

### Tyrosinase Inhibitory Assay

Tyrosinase inhibitory assays were conducted as described by Kubo and Kinst-Hori [27]. 5 mM of L-tyrosine (Sigma Chemical Co.) was used as a monophenolase substrate, and 10 mM L-DOPA (Sigma Chemical Co.) as a diphenolase substrate. After 80 μl of 0.1 M sodium phosphate buffer (pH 6.8) and 40 μl of substrate solution were preincubated at 37°C for 10 min, 40 μl of each sample and mushroom tyrosinase (40 μl, 250 U/ml) (Sigma Chemical Co.) were added, and reacted at 37°C for 20 min. Mixtures were then cooled for 5 min. Absorbances were measured using a micro-plate reader at 490 nm for monophenolase and at 475 nm for diphenolase (Tecan Sunrise). Arbutin (100 μg/ml) was used as the positive control

### Total Phenolic Compounds

Total phenolic levels were determined using the modified Folin–Ciocalteu method [28]. Briefly, 50 μl of sample, 500 μl of distilled water, and 100 μl of Folin-Ciocalteu’s phenol reagent were mixed and left to stand for 3 min in the dark. NaNO_3_ (100 μl, 10%) and 350 μl of distilled water were then added and mixtures were allowed to stand for 1 h at 25°C in the dark, and then absorbances were measured at 725 nm. A standard curve was produced to determine caffeic acid (CAE) concentrations.

### Total Flavonoid Contents

The total flavonoid contents of lemongrass extracts were measured using the modified Davis method [29]. Samples (100 μl) were mixed with 1,000 μl of 90% diethylene glycol by vigorous vortexing. NaOH solution (1 N) was then added to each sample tube and incubated at 37°C for 1 h. Absorbances were measured at 420 nm, and a standard curve was prepared to determine naringin (NAE) concentrations.

### Disc Diffusion Test

The antimicrobial effects of the 80% MeOH, n-hexane, ethyl acetate, BuOH, and water extracts were determined using the paper disc-agar plate method [30]. After repeated subculture, *C. acnes* was cultured for 24 h in brain heart infusion broth anaerobically and spread on plates at an absorbance of 0.1 at 600 nm. Extract samples (100 μl) dissolved in DMSO were loaded onto sterilized 8 mm paper-discs (ADVANTEC Toyo Roshi Kaisha., Ltd., Japan) and placed on the plates. After incubation at 37°C for 48 h under anaerobic conditions, diameters of zones of inhibition were measured.

### Relative Microbial Growth Inhibition

The relative microbial growth inhibition of lemongrass extracts was assessed using a broth micro-dilution assay [31]. *C. acnes* was cultured in brain heart infusion broth at 37°C for 18-24 h. After cultivation, *C. acnes* was adjusted to an absorbance of 0.1 at 600 nm, and then cultivated *C. acnes* (198 μl) and the serial two folded dilutions of lemongrass extracts (2 μl) were inoculated into sterile 96-well plates. DMSO was used as the negative control. After incubating under anaerobic conditions at 37°C for 24 h, relative microbial growth ratios of *C. acnes* were determined by measuring absorbance at 600 nm according to the concentration of lemongrass extracts.

### Inhibitory Assay of Bacterial Lipase Enzyme

To evaluate the inhibitory activities of bacterial lipase enzyme, modified fluorescent assay was performed [32]. *C. acnes* was activated in the BHIB medium at 37°C for 24 h. To produce lipase enzyme, activated *C. acnes* was ultrasonicated for 30 sec and then kept in ice. The ultrasonicated culture was centrifuged at 12,000 rpm for 3 min. 50 μl of supernatant, 50 μl of lemongrass sample and 100 μl of 0.1 mM 4-methyl umbelliferyl oleate (Sigma Chemical Co.) dissolved in buffer solution were mixed. Buffer solution is consisted of 13 mM Tris-HCl, 150 mM NaCl, and 1.3 mM CaCl_2_ (pH 8.0). In this test, DMSO was used as a control. The mixture was reacted at 37°C for 30 min in the presence of the light. To terminate the enzyme reaction, 200 μl of 0.1 M sodium citrate solution (pH 4.2) was added. Fluorescence was identified using a fluorescence plate reader with excitation at 355 nm and emission at 460 nm (SpectraMax Gemini EM, Molecular Devices, USA). This test was performed in triplicate.

### HPLC coupled with PDA

Lemongrass extracts were analyzed using a HPLC (high performance liquid chromatography) unit (iLC3000, Interface Engineering Co., Ltd., Korea) equipped with a photodiode array detector (PDA). The chromatograms were monitored using Clarity chromatography software (DataApex, The Czech Republic). The YMC-Triart C18 column (4.6 mm × 250 mm, i.d. 5 μm) (YMC Co., Ltd., Japan) used was equilibrated for 10 min. The mobile phase consisted of 0.1% formic acid in water (v/v) (solvent A) and acetonitrile (solvent B). The gradient condition used was as follows; 0-40 min 20→50% B and 40-50 min 50→58% B. The flow rate was 0.8 ml/min and 20 μl aliquots of samples were injected.

### LC-MS Analysis

LC-MS analysis was performed with an Acquity HSS T3 column (2.1 × 100 mm, i.d. 1.8 μm) (Waters, France), an Agilent 1290 Infinity HPLC system (Agilent, Germany), and a 6530 accurate-mass Q-TOF LC-MS system (Agilent, USA). The source parameters were as follows: gas temperature 300°C, gas flow 9 L/min, nebulizer 45 psig, sheath gas temperature 350°C, sheath gas flow 11 L/min, VCap 4000 V, and fragmentor voltage 90 V using ESI negative ([M–H]^-^) and positive ion mode ([M+H]^+^).

### Statistical Analysis

The experimental data were analyzed by one-way analysis of variance (ANOVA) in SPSS for Windows ver. 23.0 (SPSS Inc., USA). Mean and standard deviations (SDs) were determinded using Duncan’s multiple range test, and *p*-values < 0.05 were considered significant.

## Results and Discussion

### Lemongrass Extracts

Five extracts were prepared to evaluate the antioxidative, cosmeceutical, and antimicrobial properties of lemongrass. The yields of the 80% MeOH, n-hexane, EtOAc, BuOH, and water extracts were 15.36, 4.23, 13.75, 16.81, and 54.93%, respectively. Extracts yields were lower for hydrophobic than hydrophilic compounds.

### Antioxidant Activities of Lemongrass Extracts

The antioxidant activities of lemongrass extracts were assessed by determining DPPH radical scavenging, ABTS radical scavenging, and NO scavenging activities. The 80% MeOH, hexane, EtOAc, BuOH, and water extracts had DPPH radical scavenging activities of 37.17, 11.11, 58.06, 64.16, and 11.47%, respectively (Table 1), ABTS radical scavenging activities of 23.53, 9.66, 44.14, 36.68, and 18.70%, respectively, and NO scavenging activities of 35.88, 37.45, 41.08, 38.63, and 18.04%, respectively. TEAC and NO radical scavenging activity results showed the EtOAc extract had significantly higher antioxidant activity than other fractions, whereas the BuOH fraction had the highest DPPH radical scavenging activity.

In a previous study, lemongrass extracts were found to scavenge the superoxide anion and inhibit the lipoperoxidation and decolorization of DPPH [6]. In general, conventional hydroalcoholic extraction is a better means of obtaining lemongrass extracts containing greater amounts of bioactive compounds and the use of an extraction temperature of 60°C produces extracts with higher antioxidant activity [33]. An aqueous ethanol extract was reported to decrease reactive oxygen species production and lipid peroxidation and to increase superoxide dismutase activity and glutathione levels [34]. Recently, essential oil of lemongrass was reported to be an antioxidant as determined by the DPPH scavenging test. The results obtained showed that both leaf and stalk extracts possessed dose-dependent radical scavenging [35]. Various antioxidative properties such as DPPH radical, nitric oxide, and ABTS scavenging activity and reducing power provide a basis for selectively identifying appropriate species for further characterization and for evaluating antioxidant properties of active components and determining total polyphenol and flavonoid contents [36].

### Anti-aging and Whitening Activities of Lemongrass Extracts

Elastase inhibitory activities of the MeOH, n-hexane, EtOAc, BuOH, and water extracts were 44.23%, 49.05%, 60.78%, 48.14%, and 35.81% at an extract concentration of 200 μg/ml (Table 2). Ursolic acid (the positive control) inhibited elastase activity by 70.64% at 100 μg/ml. The EtOAc extract had highest elastase inhibitory activity among various extracts. Elastase breaks down elastin (a fibrous protein) and can damage skin and cause wrinkles [37]. Some studies suggest that elastase-producing strains of *Cutibacterium acnes* in hair follicles are associated with the pathogenesis [38].

The MeOH, n-hexane, EtOAc, BuOH, and water extracts of lemongrass at 33 μg/ml inhibited collagenase by 58.84%, 72.89%, 75.06%, 64.50%, and 63.25%, respectively (Table 2). On the other hand, epigallocatechin gallate (the positive control) at 16.5 μg/ml inhibited collagenase by 65.45%. Thus the EtOAc and hexane extracts most inhibited collagenase. This enzyme breaks down collagen, which is largely responsible for skin barrier properties, and causes wrinkling, and its activity is increased by aging [39].

The tyrosinase inhibitory activities of lemongrass extracts were investigated using L-tyrosine and L-DOPA as substrates. In the case of monophenolase inhibitory activity, the BuOH extract had the greatest inhibitory effect of 36.92%, whereas the hexane extract inhibited diphenolase inhibitory activity most (25.16%). On the other hand, arbutin (the positive control) inhibited monophenolase activity by 33.03% and diphenolase by 23.50% at 100 μg/ml. Tyrosinase catalyzes two distinct reactions of melanin synthesis, the hydroxylation of monophenols and the conversion of *O*-diphenols to the corresponding *O*-quinones. In addition, tyrosinase inhibitors are becoming increasingly important in the pharmaceutical and cosmetic fields as potential treatments for hyperpigmentation [27].

### Antimicrobial Activities of Lemongrass Extracts

To explore the antimicrobial activities of the five lemongrass extracts, we used a paper disc diffusion assay (Table 3). Diameters of inhibitory zones against *Cutibacterium acnes* for the 80% MeOH, n-hexane, EtOAc, BuOH, and water extracts were 13.97, 17.02, 20.65, 14.19, and 8.00 mm at 5 mg/disc, respectively, which showed the EtOAc extract was most effective. Many herbs have exhibit antimicrobial activities against *C. acnes*, such as *Hemidesmus indicus* and *Euphorbia hirta*, which both produced inhibitory zones of diameter 13 mm against *C. acnes* at a concentration of 100 μg/ml [40]. In the present study, the EtOAc extract of lemongrass, which had the greatest effect, produced an inhibitory zone of diameter against *C. acnes* of 13.36 mm at 0.5 mg/disc (10 μg/ml).

The relative microbial growth inhibition ratios of lemongrass extracts toward *C. acnes* were also examined. Each of the five extracts was prepared at the presence of 500 μg/ml and then added in the culture broth containing activated *C. acnes*. A control containing only DMSO was also prepared. After incubating under anaerobic conditions for 24 h, relative growths were assessed spectrophotometrically. Relative growth inhibition ratios of the 80% MeOH, hexane, EtOAc, BuOH, and water extracts were 2.36, 15.49, 34.20, 7.55, and 0.39%, respectively. Consequentially, the EtOAc extract most inhibited microbial growth. This is the first report that lemongrass extracts suppress the growth of *C. acnes*.

The lipase inhibitory activity against *C. acnes* were analyzed as shown in Table 4. Lipase, one of the mainly virulence factors of the *C. acnes*, hydrolyzes triglycerides to release free fatty acids. Lipase overexpression improves follicular development and causes acne vulgaris [41]. Therefore, the less lipase is secreted, the less acne is caused. Among lemongrass extracts, EtOAc extract showed the highest lipase inhibitory activity. Lipase inhibitory activity of lemongrass 80% MeOH, hexane, EtOAc, BuOH, and Water extracts were 10.02%, 33.20%, 74.08%, 17.43%, and 21.30% in the presence of 10 μg/ml.

### Total Phenolic and Flavonoid Contents of Lemongrass Extracts

Phenolic compounds are commonly found in plants and have been reported to have many biological effects, which include anticancer, antioxidant, and anti-inflammatory properties [42]. Total phenolic contents of lemongrass extracts are expressed as mg of caffeic acid equivalents per 1 g of sample. As shown in Table 5, the EtOAc extract contained more phenolics (total phenolic content 132.3 mg CAE/g), and the 80% MeOH, hexane, butanol, and water extracts had phenolic contents of 90.25, 79.27, 108.78, and 63.20 mg CAE/g, respectively. In a previous study, lemongrass was found to have a phenolic content of 662.0 mg GAE/100 g) [5].

Flavonoids have two or more aromatic rings, which contain at least one hydroxyl group and are connected by a carbon bridge [43]. It has been reported that flavonoids have anti-inflammatory, antioxidative, anti-aging, antimicrobial, and anti-cancer effects [44]. We found the total flavonoid content of the EtOAc extract had the highest flavonoid content at 132.31 mg NE/g whereas those of the methanol, hexane, butanol, and water extracts were 23.07, 12.12, 76.40, and 18.07 mg NE/g, respectively. In another study, lemongrass (300.5 mg CAE/100 g) was reported to have a moderate flavonoid content (300.5 mg CAE/100 g) [5]. We found the phenolic and flavonoid contents of the EtOAc extract were around 13.2% and 10.4% by weight and significantly affected antioxidant activity, as determined by DPPH, TEAC, and NO assays.

### Identification of Biological Compounds in Lemongrass Extracts

**Fig. 1:**
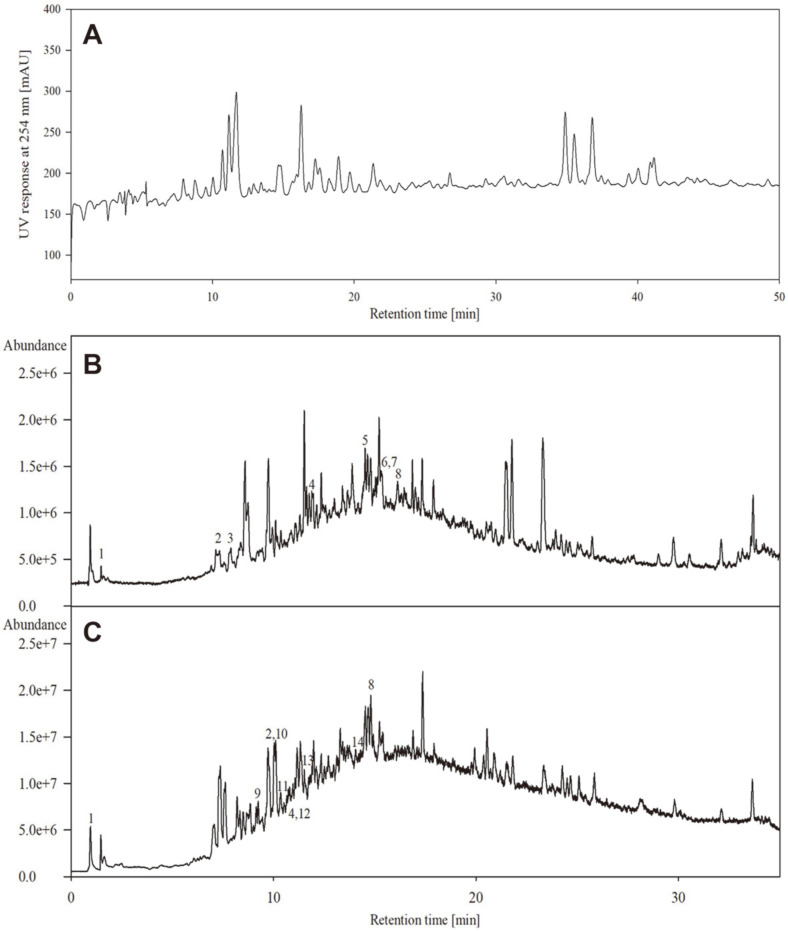
(**A**) HPLC chromatogram (**B**) UHPLC-ESI-MS chromatographic profile of the lemongrass EtOAc fraction in negative ion mode and (**C**) UHPLC-ESI-MS chromatographic profile of lemongrass EtOAc fraction in the positive ion mode.

The EtOAc extract was analyzed by HPLC equipped with a photodiode array detector (PDA) (Fig. 1A). Many peaks were detected at 254 nm. HPLC analysis showed the EtOAc extract contained the main free radical scavengers and antioxidants in lemongrass.

To analyze the compounds in EtOAc extract with the highest antioxidant and antimicrobial efficacies, we used HPLC PDA (Fig. 1A), and to analyze phenolic acid levels, we used LC-ESI-MS in negative ([M–H]^-^) and positive mode ([M+H]^+^).

In negative ion mode, phenolic acids in the EtOAc extract were cinnamic acid, salicylic acid, protocatechuic acid, ferulic acid, chrysoriol 7-*O*-glucoside, catechin, isovitexin, and tricin (Fig. 1B), and positive ion mode showed it contained cinnamic acid, caffeic acid, salicylic acid, p-hydroxybenzoic acid, gallic acid, ferulic acid, isovitexin, luteolin, catechin, and tricin (Fig. 1C). In addition, in negative ion mode, the percentage peak areas of the phenolic acids, namely cinnamic acid in peak 1, salicylic acid in peak 2, protocatechuic acid in peak 3, ferulic acid in peak 4, chrysoriol 7-*O*-glucoside in peak 5, catechin in peak 6, isovitexin in peak 7, and tricin in peak 8 were 2.0, 1.6, 0.7, 1.3, 3.2, 4.5, 4.5, and 1.1 %, respectively. On the other hand, in positive ion mode, the percentage peak areas of the phenolic acids, namely cinnamic acid in peak 1, caffeic acid in peak 9, salicylic acid in peak 2, p-hydroxybenzoic acid in peak 10, gallic acid in peak 11, ferulic acid in peak 4, isovitexin in peak 12, luteolin in peak 13, catechin in peak 14, and tricin in peak 8 were 2.3, 0.4, 2.5, 2.5, 0.4, 0.5, 0.5, 0.6, 0.4, and 2.5 %, respectively. Many of these compounds have been reported to have antioxidant activities [45] and are used as cosmetic ingredients. In particular, protocatechuic acid is a potential skin antiseptic agent that demonstrated dose-dependent skin penetration and antimicrobial activity against *C. acnes* in mouse skin [46].

In this study, we evaluated the antioxidative, cosmeceutical, and antimicrobial properties of lemongrass extracts and identified the biologically active compound in the EtOAc extract. Based on our results, the EtOAc extract of lemongrass is promising in a potential skin care cosmeceutical active against *Acne vulgaris*. However, further studies are needed to evaluate the anti-inflammatory effects in the human keratinocyte cell line and antibacterial mechanisms of EtOAc extract of lemongrass. In addition, we need to investigate the identified active components in this study associated with in vitro and in vivo cosmetic effects and antibacterial activity against *C. acnes* and thus conduct more focused studies in the path of cosmetic development for Acne vulgaris.

## Figures and Tables

**Table 1:** Antioxidant activities of lemongrass extracts.

Extracts	DPPH (%)^1^	TEAC (%)^2^	Nitric oxide (%)^3^
80% MeOH	37.17 ± 0.88^c^	23.53 ± 0.63^c^	35.88 ± 1.56^c^
Hexane	11.11 ± 0.62^d^	9.66 ± 0.30^e^	37.45 ± 1.03^bc^
EtOAc	58.06 ± 1.08^b^	44.14 ± 0.30^a^	41.08 ± 0.45^a^
BuOH	64.16 ± 1.64^a^	36.68 ± 1.17^b^	38.63 ± 1.70^b^
Water	11.47 ± 2.48^d^	18.70 ± 1.22^d^	18.04 ± 1.51^d^
L-Ascorbic acid	97.49 ± 0.62	99.09 ± 0.08	

Values are mean ± S.D.

^1^Sample was 50 μg/ml

^2^Sample was 10 μg/ml

^3^Sample was 50 μg/ml

^a-e^Values are significantly different as determined by Duncan’s multiple test (*p* < 0.05)

**Table 2:** Anti-anging and whitening activities of lemongrass extracts.

Extracts	Elastase inhibition (%)^1^	Collagenase inhibition (%)^2^	Tyrosinase inhibition	

			Monophenolase inhibition (%)^3^	Diphenolase inhibition (%)^3^
80% MeOH	44.23 ± 0.52^c^	58.84 ± 3.93^c^	26.01 ± 1.19^b^	19.17 ± 0.49^bc^
Hexane	49.05 ± 1.20^b^	72.89 ± 1.97^a^	25.41 ± 0.68^b^	25.16 ± 1.61^a^
EtOAc	60.78 ± 0.46^a^	75.06 ± 0.64^a^	26.16 ± 0.26^b^	21.22 ± 2.79^ab^
BuOH	48.14 ± 0.66^b^	64.50 ± 1.12^b^	36.92 ± 1.70^a^	22.17 ± 1.23^ab^
Water	35.81 ± 2.87^d^	63.25 ± 1.12^b^	7.17 ± 1.55^c^	15.86 ± 3.28^c^
Positive control	70.64 ± 0.55^4^	65.45 ± 4.92^5^	33.03 ± 1.13^6^	23.50 ± 1.60^6^

Values are mean ± S.D.

^1,3^Sample was 200 μg/ml

^2^Sample was 33 μg/ml

^4^Ursolic acid was 100 μg/ml

^5^Epigallocatechin gallate was 16.5 μg/ml

^6^Arbutin was 100 μg/ml

^a-d^Values are significantly different as determined by Duncan’s multiple test (*p* < 0.05)

**Table 3:** Disc diffusion test for antimicrobial activity against *Cutibacterium acnes*.

		mg/disc	Control	80% MeOH	Hexane	EtOAc	BuOH	Water
Clear zones on plate (mm)	KCTC 3314 (+)*	0.5	8.0 ± 0.1^c^	N.D.^1^	11.30 ± 0.4^b^	13.36 ± 0.4^a^	N.D.	N.D.
		1	8.0 ± 0.1^c^	N.D.	13.90 ± 0.1^b^	15.13 ± 0.3^a^	N.D.	N.D.
		5	8.0 ± 0.1^d^	13.97 ± 0.1^c^	17.02 ± 0.2^b^	20.65 ± 0.2^a^	14.19 ± 0.2^c^	N.D.
		10	8.0 ± 0.1^d^	15.26 ± 0.1^d^	18.43 ± 0.3^b^	21.79 ± 0.2^a^	17.70 ± 0.4^c^	N.D.

Values are mean ± S.D.

^1^Not detected

^a-d^Values are significantly different as determined by Duncan’s multiple test (*p*<0.05)

*KCTC 3314 (*C. acnes*)

**Table 4:** Lipase inhibitory activity of lemongrass extracts toward *Cutibacterium acnes*.

	Lipase inhibitory activity (%)^1^
80% MeOH	10.02 ± 1.18^e^
Hexane	33.20 ± 2.82^b^
EtOAc	74.08 ± 0.61^a^
BuOH	17.43 ± 1.43^d^
Water	21.30 ± 1.65^c^

Values are mean ± S.D.

^1^Sample was 10 μg/ml

^a-e^Values are significantly different as determined by Duncan’s multiple test (*p* < 0.05)

**Table 5:** Total polyphenol and flavonoid contents of lemongrass extracts.

	Total phenolic compound^1^	Total flavonoid content^2^
80% MeOH	90.25 ± 0.93^c^	28.07 ± 2.86^c^
Hexane	79.27 ± 0.66^d^	12.12 ± 0.41^e^
EtOAc	132.31 ± 0.93^a^	104.50 ± 1.43^a^
BuOH	108.78 ± 2.72^b^	74.40 ± 0.41^b^
Water	63.20 ± 1.81^c^	18.07 ± 1.89^d^

Values are mean ± S.D.

^1^Caffeic acid was used as a standard

^2^Naringin was used as a standard

^a-e^Values are significantly different as determined by Duncan’s multiple test (*p* < 0.05)

## References

[1] Yi MR, Kang CH, Bu HJ (2017). Acetic acid fermentation properties and antioxidant activity of lemongrass vinegar. Kor. J. Food Preserv..

[2] Chae IG, Kim HJ, Yu MH, Kim HI, Lee IS (2010). Antioxidant and antibacterial activity of commercially available herbs in Korean markets. J. Kor. Soc. Food Sci. Nutr..

[3] Tzortzakis NG, Economakis CD (2007). Antifungal activity of lemongrass (*Cympopogon citratus* L.) essential oil against key postharvest pathogens. Innov. Food Sci. Emerg. Technol..

[4] Maheswari RU, EuginAmala V (2015). Analyzing and determining the activity of antimicrobial, functional group and phytochemicals of *Cymbopogon citratus* using well Diffusion, FT-IR and HPLC. Alcohol.

[5] Yoo KM, Lee CH, Lee H, Moon B, Lee CY (2008). Relative antioxidant and cytoprotective activities of common herbs. Food Chem..

[6] Cheel J, Theoduloz C, Rodríguez J, Schmeda-Hirschmann G (2005). Free radical scavengers and antioxidants from Lemongrass (*Cymbopogon citratus* (DC) Stapf.). J. Agric. Food Chem..

[7] Ming LC, Figueiredo RO, Machado SR, Andrade RMC (1995). Yield of essential oil of and citral content in different parts of lemongrass leaves (*Cymbopogon citratus* (dc) stape) poaceae. Acta Hortic..

[8] Miean KH, Mohamed S (2001). Flavonoid (myricetin, quercetin, kaempferol, luteolin, and apigenin) content of edible tropical plants. J. Agric. Food Chem..

[9] Khadri A, Neffati M, Smiti S, Falé P, Lino ARL, Serralheiro MLM (2010). Antioxidant, antiacetylcholinesterase and antimicrobial activities of *Cymbopogon schoenanthus* L. Spreng (lemon grass) from Tunisia. LWT-Food Sci. Technol..

[10] Syed M, Khalid MR, Chaudhary FM (1990). Essential oils of Gramineae family having antibacterial activity. Part-1.(*Cymbopogon citratus*, *C. martinii* and *C. jawarancusa* oils). Pakistan J. Sci. Ind. Res..

[11] Ibrahim D (1992). Antimicrobial activity of the essential oil of the local serai, *Cymbopogon citratus*. J. Biosci..

[12] Baratta MT, Dorman HD, Deans SG, Figueiredo AC, Barroso JG, Ruberto G (1998). Antimicrobial and antioxidant properties of some commercial essential oils. Flavour Fragr. J..

[13] Tanase C, Bara CI, Popa VI (2015). Cytogenetical effect of some polyphenol compounds separated from industrial by-products on maize (*Zea Mays* L.) plants. Cellul. Chem. Technol..

[14] Tepe B., Sokmen M., Akpulat H. A., Sokmen A. (2006). Screening of the antioxidant potentials of six salvia species from Turkey. Food Chem..

[15] Sujarwo W, Keim AP, Watson RR, Preedy VR (2019). *Spondias pinnata* (L. f.) Kurz. (Anacardiaceae): Profiles and Applications to Diabetes. Bioactive Food as Dietary Interventions for Diabetes.

[16] Beckman JS, Koppenol WH (1996). Nitric oxide, superoxide, and peroxynitrite: the good, the bad, and ugly. Am. J. Physiol. Cell Physiol..

[17] Pacher P, Beckman JS, Liaudet L (1996). Nitric oxide and peroxynitrite: in health and disease. Physiol. Rev..

[18] Shin KH, Lee HS, Kim KC (2008). Sebum creation and acne. J. Skin Barrier Res..

[19] Dréno B, Pécastaings S, Corvec S, Veraldi S, Khammari A, Roques C (2018). *Cutibacterium acnes* (*Propionibacterium acnes*) and acne vulgaris: a brief look at the latest updates. J. Eur. Acad. Dermatol. Venereol..

[20] Rosenthal M, Goldberg D, Aiello A, Larson E, Foxman B (2011). Skin microbiota: microbial community structure and its potential association with health and disease. Infect. Genet. Evol..

[21] Christensen GJM, Brüggemann H (2014). Bacterial skin commensals and their role as host guardians. Benef. Microbes.

[22] Blois MS (1958). Antioxidant determinations by the use of a stable free radical. Nature.

[23] Re R, Pellegrini N, Proteggente A, Pannala A, Yang M, Rice-Evans C (1999). Antioxidant activity applying an improved ABTS radical cation decolorization assay. Free Rad. Biol. Med..

[24] Jaiswal YS, Tatke PA, Gabhe SY, Ashok V (2010). Antioxidant activity of various extracts of leaves of *Anacardium occidentale* (cashew). Res. J. Pharm. Biol. Chem. Sci..

[25] Cannell JJ (1988). Nationally normed elementary achievement testing in America's public schools: how all 50 states are above the national average. Educ. Meas..

[26] Wünsch E, Heidrich HG (1963). Zur quantitativen bestimmung der kollagenase. Hoppe-Seylers Z. Physiol..

[27] Kubo I, Kinst-Hori I (1999). Flavonols from saffron flower: tyrosinase inhibitory activity and inhibition mechanism. J. Agric. Food Chem..

[28] Kumaran A, Karunakaran RJ (2007). In vitro antioxidant activities of methanol extracts of five *Phyllanthus* species from India. LWTFood Sci. Technol..

[29] Chang CC, Yang MH, Wen HM, Chern JC (2002). Estimation of total flavonoid content in propolis by two complementary colorimetric methods. J. Food Drug Anal..

[30] De Beer EJ, Sherwood MB (1945). The paper-disc agar-plate method for the assay of antibiotic substances. J. Bacteriol..

[31] Hammer KA, Carson CF, Riley TV (1996). Susceptibility of transient and commensal skin flora to the essential oil of *Melaleuca alternifolia* (tea tree oil). Am. J. Infect. Control.

[32] Inui S, Aoshima H, Ito M, Kokubo K, Itami S (2012). Inhibition of sebum production and *Propionibacterium acnes* lipase activity by fullerenol, a novel polyhydroxylated fullerene: potential as a therapeutic agent for acne. J. Cosmet. Sci..

[33] Boeira CP, Piovesan N, Soquetta MB, Flores DCB, Lucas BN, Rosa CSD (2018). Extraction of bioactive compounds of lemongrass, antioxidant activity and evaluation of antimicrobial activity in fresh chicken sausage. Cienc. Rural.

[34] Tiwari M, Dwivedi UN, Kakkar P (2010). Suppression of oxidative stress and pro-inflammatory mediators by *Cymbopogon citratus* D.Stapf extract in lipopolysaccharide stimulated murine alveolar macrophages. Food Chem. Toxicol..

[35] Mirghani MES, Liyana Y, Parveen J (2012). Bioactivity analysis of lemongrass (*Cymbopogan citratus*) essential oil. Int. Food Res. J..

[36] Choi YJ, Hwang KH (2013). Radical scavenging activities of fruits of *Crataegus pinnatifida* BUNGE major.from Korea. Nat. Prod. Sci..

[37] Antonicelli F, Bellon G, Debelle L, Hornebeck W (2007). Elastin‐elastases and inflamm‐aging. Curr. Top. Dev. Biol..

[38] Christensen GJM, Brüggemann H (2014). Bacterial skin commensals and their role as host guardians. Benef. Microbes.

[39] Karim AA, Azlan A, Ismail A, Hashim P, Abd Gani SS, Zainudin BH (2014). Phenolic composition, antioxidant, anti-wrinkles and tyrosinase inhibitory activities of cocoa pod extract. BMC Complement. Altern. Med..

[40] Kumar GS, Jayaveera KN, Kumar CK, Sanjay UP, Swamy BM, Kumar DV (2007). Antimicrobial effects of Indian medicinal plants against acne-inducing bacteria. Trop. J. Pharm. Res..

[41] Unno M, Cho O, Sugita T (2017). Inhibition of *Propionibacterium acnes* lipase activity by the antifungal agent ketoconazole. Microbiol. Immunol..

[42] El Gharras H (2009). Polyphenols: food sources, properties and applications-a review. Int. J. Food Sci. Technol..

[43] Peisner‐Feinberg ES, Burchinal MR, Clifford RM, Culkin ML, Howes C, Kagan SL (2001). The relation of preschool child‐care quality to children's cognitive and social developmental trajectories through second grade. Child Dev..

[44] Zhang Y, Shen JF, Yu ZY, Lu BY, Lou DD (2004). Primary studies on bamboo leaf flavonoids used as anti-aging factor for skin protection. Chem. Ind. Forest Prod..

[45] Sarker U, Oba S (2020). Polyphenol and flavonoid profiles and radical scavenging activity in leafy vegetable *Amaranthus gangeticus*. BMC Plant Biol..

[46] Jalali O, Best M, Wong A, Schaeffer B, Bauer B, Johnson L (2020). Protocatechuic acid as a topical antimicrobial for surgical skin antisepsis. JB JS Open Access.

